# Isolation and Characterization of *Globisporangium glomeratum* (syn. *Pythium glomeratum*) from Declining Holm Oak in a Historical Garden

**DOI:** 10.3390/pathogens14100960

**Published:** 2025-09-23

**Authors:** Anna Maria Vettraino, Michele Narduzzi, Chiara Antonelli

**Affiliations:** Department for Innovation in Biological, Agro-Food and Forest Systems (DIBAF), University of Tuscia, 01100 Viterbo, Italy; michele.narduzzi@unitus.it (M.N.); chiara.antonelli@unitus.it (C.A.)

**Keywords:** *Globisporangium glomeratum*, *Quercus ilex*, urban tree, historical gardens, *Phytophthora cinnamomi*, urban green areas

## Abstract

*Pythium*-like organism species are widespread soilborne oomycetes known to cause root diseases in a wide range of plant hosts. However, their involvement in the decline of woody species in historical and urban gardens has received limited attention. This study reports the isolation and identification of a *Pythium*-like organism from declining *Quercus ilex* specimens in a historical garden, where affected trees showed symptoms of root rot and sucker dieback. Integration of morphological observations and molecular analyses of ITS, LSU, and Cox II sequences confirmed the identity of the isolates as *Globisporangium glomeratum* (formerly *Pythium glomeratum*). Pathogenicity tests confirmed the aggressiveness of these isolates on *Q. ilex* seedlings, resulting in significant reductions in plant height and shoot and root biomass. The detection of *G. glomeratum* in the soil of a historical garden underscores the risk of its unintentional dissemination through nursery stock or soil movement, particularly in urban settings where plant replacement is frequent. This is the first report of *G. glomeratum* as a pathogen of *Q. ilex*, emphasizing the importance of phytosanitary monitoring in culturally and ecologically valuable green spaces.

## 1. Introduction

*Quercus ilex* (holm oak) is an evergreen oak often associated with the Mediterranean landscape and mythology. For instance, it was regarded as an earthly symbol of Zeus’s power and regarded as sacred by the Romans in honor of Jupiter. In urban areas, *Q. ilex* is more than just a decorative element, supporting environmental and human health and enhancing urban aesthetics. Therefore, together with other tree species characterizing Mediterranean urban areas and historical villas, i.e., *Platanus* trees, *Q. ilex* reflects a design philosophy that harmonizes aesthetic considerations, ecological functionality, and symbolic meaning, integrating natural elements into the cultural and spiritual identity of cities and historical gardens [1,2]. For this reason, the conservation of these tree species is essential not only for maintaining biodiversity and ecological integrity but also for safeguarding cultural heritage. The vitality of trees in urban areas, including historical gardens, is increasingly challenged by climatic factors and anthropogenic stressors, such as a limited rooting volume imposed by stone paving or construction and maintenance practices [3,4]. Of particular concern is the emergence and spread of pathogenic members of the class Oomycetes, such as *Pythium* and *Phytophthora* species. These microorganisms are commonly reported in urban areas to threaten tree health, seriously diminishing their capacity to provide essential ecosystem services [5,6,7,8]. Their capacity to withstand a broad spectrum of abiotic stresses, including temperature extremes and pH shifts, and to be advantaged by water availability makes these pathogens particularly resilient and facilitates their accidental spread into urban ecosystems via contaminated plant material [9,10,11,12,13].

In the summer of 2024, wilting and dieback of two mature *Q. ilex* trees were observed in a historical garden in Viterbo, Italy. In order to determine the etiology of the observed symptoms, this study aimed to assess whether soilborne pathogens might be involved in tree decline.

## 2. Materials and Methods

A survey was conducted in 2024 in a historical park in Central Italy (Viterbo, Italy), where suckers of two holm oak trees, more than 60 years old, showed leaf chlorosis and suddenly died. Pollons were around the tree stumps. Based on the gardener’s observations, the plants started dying over the past three years, showing thinning of the foliage followed by death. The exact age of the plants is unknown, and the stump diameter was 125 cm. These trees were probably part of the historical context of a 16th-century formal garden, which has been preserved since then.

The area studied is composed of *Platanus orientalis*, *Buxus sempervirens*, and floral crops, which were recently renewed using plants from a local nursery. Soil and root samples were randomly collected from the rhizosphere of 4 *Q. ilex* trees, 2 symptomatic and 2 asymptomatic specimens, and one *B. sempervirens* plant which showed decline symptoms.

Samples were processed by the baiting assay described by Antonelli et al. [5] with small changes. Specifically, 200 mL of soil per sample were placed in plastic containers and flooded with 2 L of distilled water. After 24 h, any debris and soil particles on the surface of the water were removed, and young leaves of *Q. ilex*, *Q. robur*, and *Sambucus nigra* leaves were used as bait to capture oomycete zoospores. When lesions appeared, leaves were dried on paper towels, cut into small sections (2 mm^2^), and placed on PARP-V8 selective medium (V8 juice, 50 mL/L; CaCO_3_, 3.5 g/L; pimaricin, 5 mg/L; ampicillin, 250 mg/L; rifampicin, 10 mg/L; pentachloronitrobenzene, 50 mg/L; and agar, 15 g/L (Oxoid Ltd., Basingstoke, UK). Hyphal tips from the obtained colonies were subcultured on Carrot Agar (CA; carrots, 200 g/L and agar, 15 g/L) at 22 °C in the dark. Sporangia production was induced by feeding 7-day old colonies on CA with sterile distilled water, soil extract, and carrot juice. Colonies were grouped according to their morphology and asexual structures on CA and Potato Dextrose Agar (PDA; 39 g/L; Oxoid Ltd., Basingstoke, UK), observed after 7 days of incubation. The isolate N54 was chosen as a representative of the morphotypes obtained. It was stored on PDA slants in a culture collection of Professor Anna Maria Vettraino (Laboratory of Plant Protection—DIBAF, University of Tuscia, Viterbo, Italy). The identity of the isolate N54 was confirmed by molecular analysis. Genomic DNA was extracted using the NucleoSpin kit (Macherey-Nagel GmbH & Co., Duren, Germany), and ITS, LSU, and Cox II gene regions were amplified [14,15]. The obtained sequences were blasted using BLASTN and were aligned with the closely associated reference sequences derived from the GenBank database. A phylogenetic tree was constructed using the Neighbor-Joining method with the Kimura 2-parameter model using the MEGA 11 software [16,17]. Bootstrap analysis was based on 1000 replications. The sequences were deposited in GenBank under the accession numbers listed in Table 1.

A pathogenicity test was performed on 3-month holm oak seedlings using the isolate N54 and *Phytophthora cinnamomi* Ph28, for comparison. The pathogens were grown on sterilized millet seeds moistened with V8 broth, as described by Antonelli et al. [5]. One-week-old inoculated millet was mixed into the potting soil at a rate of 1% (*v*/*v*) to inoculate *Q. ilex* seedlings. The inoculum was rinsed with deionized water to remove excess nutrients immediately before use. After inoculation, pots were flooded for 24 h to promote *Phytophthora* sporulation and zoospore release. The flooding was repeated after 15 days for 48 h. A total of 12 seedlings/treatments were used. Control seedlings were not inoculated.

Pots were arranged in a randomized complete block design on benches of a greenhouse at 22 °C. The experiment lasted two months. At the end of the experiment, the presence of pathogens was confirmed by their re-isolation from 5 small root portions per seedling from each pot on CA.

At the end of the experiment, the seedlings’ height as well as the fresh and dry weights of both shoots and roots were recorded.

Data were checked for normality by the Shapiro–Wilk test and then subjected to analysis of variance (ANOVA) using the GraphPad Prism software (version 8.0.1, San Diego, CA, USA). Significant differences among mean values were determined using Tukey’s Test at a significance level of 5%, assuming *p* < 0.05 as a significant value.

## 3. Results and Discussion

**Figure 1:**
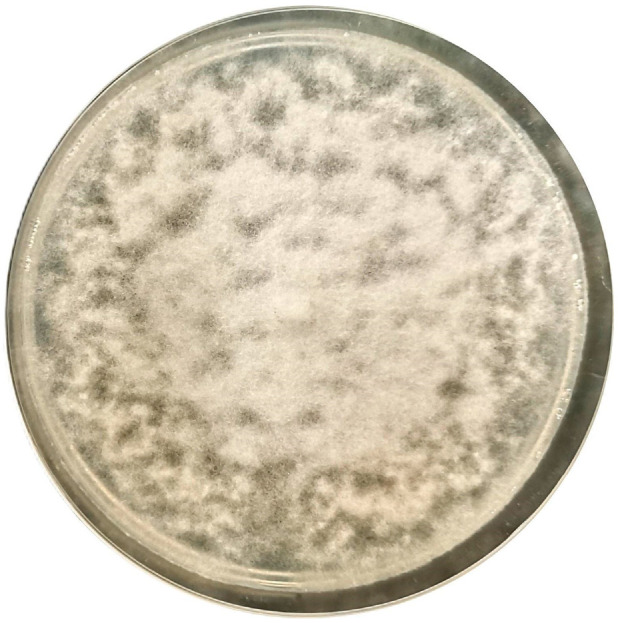
*Globisporangium glomeratum* N54 isolate grown on PDA medium after 7 days of incubation in 25 °C.

Oomycete-like isolates were obtained only from the rhizosphere samples collected around symptomatic *Q. ilex* trees. All 11 isolates obtained displayed a chrysanthemum pattern on PDA, with an average growth at 25 °C of 13 mm per day (Figure 1).

**Figure 2:**
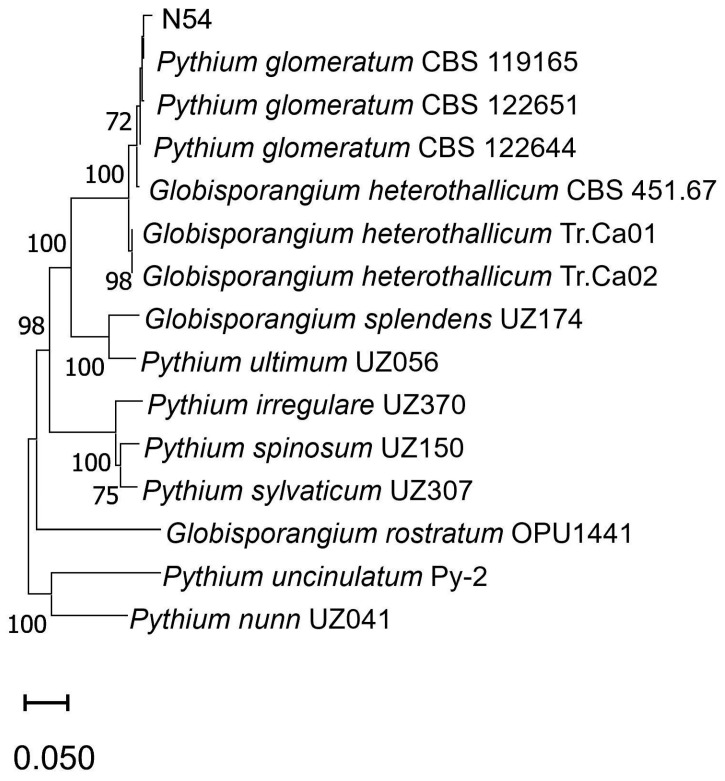
Neighbor-Joining phylogenetic tree of *G. glomeratum* N54 based on a combined matrix of ITS, LSU, and Cox II genes and its closest GenBank relatives (accession numbers in parentheses). Bootstrap support values ≥ 50% (1000 replicates) are shown at the nodes.

The oomycete N54 did not produce sporangia and zoospores, even after prolonged flooding with sterile distilled water, soil extract, and carrot juice. Oogonia were spherical and rarely elongated, with one to six antheridial branches per oogonium. DNA sequence analysis confirmed that the isolate N54 belonged to the species *Globisporangium glomeratum* (Figure 2).

The clustering of *G. glomeratum* N54 together with *P. glomeratum* is unsurprising given the evidence that the traditional genus *Pythium* is polyphyletic. In 2010, molecular phylogenetic analyses led to the reclassification of several species formerly assigned to the genus *Pythium*, which are now placed under the newly established genus *Globisporangium* [18,19].

*Pythium glomeratum* was first isolated from soil samples in France in 1992 but was incorrectly identified as *P. heterothallicum* [20]. *Pythium glomeratum* has proven to cause soybean damping-off and root rot [21,22], while *P. heterothallicum* affects mainly horticulture and floral crops [23,24]. In 2016, *P. glomeratum* and *P. heterothallicum* were isolated from diseased Aleppo pine seedlings in forest nurseries in Algeria [25]. Nevertheless, both *P. glomeratum* and *P. heterothallicum* have never been associated with diseased quercus trees.

**Figure 3:**
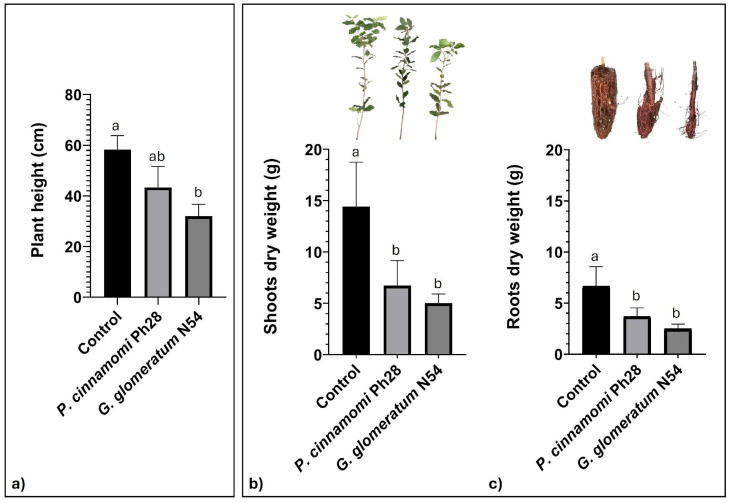
Plant height (**a**) and dry weight of shoots (**b**) and roots (**c**) of *Q. ilex* after two months of growth in uninfected soil (control) and in soil infected by *P. cinnamomi* Ph28 and *G. glomeratum* N54. Data are means ± standard errors; different alphabets at the top of error bars of means represent significant differences (ANOVA, *p* < 0.05).

Both *P. cinnamomi* Ph28 and *G. glomeratum* N54 have proven to be pathogenic on *Q. ilex* seedlings. All inoculated plants developed severe symptoms of wilting (80%) and final death (20%) within four weeks after the inoculation and a significant reduction in shoot and root biomass (Figure 3). *Phytophthora cinnamomi* Ph28 was shown to be less aggressive than *G. glomeratum* N54. The control plants remained symptomless. Both the pathogens were successfully re-isolated from necrotic root tissues of all inoculated plants, thus fulfilling Koch’s postulates. No pathogen colonies were isolated from the control plants.

Overall, the results of this study report, for the first time, root rot disease of *Q. ilex* caused by *G. glomeratum*. This is a critical concern because holm oak naturally produces suckers, particularly from the base of the trunk or roots, as a strategy for renewal and regeneration. This regenerative capacity is crucial for the long-term survival and resilience of oak trees, especially in the Mediterranean climate, where they are frequently exposed to drought, fire, and other stressors. Therefore, the report of oak death caused by *G. glomeratum* raises significant worries, as it highlights the potential for this pathogen to emerge as a serious widespread threat, especially in urban areas where it could be introduced through plant renovation plans. Although in this study *G. glomeratum* was not isolated from ornamental plants recently introduced in the garden, *P. glomeratum* has been formally detected in nurseries [25]. This is particularly relevant given that the trade of plants and seeds is well known to facilitate the spread of plant pathogens [26,27,28].

One more aspect that heightens the risk associated with the potential dissemination of *G. glomeratum* is its significantly greater aggressiveness compared to *P. cinnamomi*, one of the most threatening pathogens affecting *Q. ilex* and contributing to tree mortality in Mediterranean ecosystems and nurseries [29,30,31,32].

The findings of this study emphasize the urgent need for ongoing monitoring of tree health, particularly in historical gardens, to ensure their preservation and to prevent pathogen spread within culturally and ecologically valuable landscapes. Further research is needed to understand how environmental conditions, such as changes in temperature, humidity, and soil compositions, affect both tree susceptibility and *G. glomeratum* aggressiveness.

Understanding these interactions is essential for developing effective and site-specific management strategies aimed at preserving tree health and ensuring the long-term functionality of urban green spaces.

## Figures and Tables

**Table 1:** GenBank accession numbers for DNA sequences used in this study.

		GenBank Accession Numbers		
Species	Isolate	ITS	LSU	Cox II
In this study	N54	PX275941	PX282578	PX308648
*Globisporangium heterothallicum*	CBS 451.67	AB507409	AB513045	AB513045
*G. heterothallicum*	Tr.Ca01	MT039879	MT039885	MT039885
*G. heterothallicum*	Tr.Ca02	MT039880	MT039886	MT039886
*G. rostratum*	OPU1441	AB468775	AB468713	AB468713
*G. splendens*	UZ174	AB468778	AB468716	AB468716
*Pythium glomeratum *	CBS 122644	HQ643542	HQ665097	HQ665097
*P. glomeratum*	CBS 119165	HQ643544	HQ665085	----
*P. glomeratum*	CBS 122651	HQ643541	HQ665104	----
*P. irregulare*	UZ370	AB468770	AB468706	AB468706
*P. nunn*	UZ041	AB468771	AB468709	AB468709
*P. spinosum*	UZ150	AB468776	AB468714	AB468714
*P. sylvaticum*	UZ307	AB468779	AB468717	AB468717
*P. ultimum*	UZ056	AB468781	AB468719	AB468719
*P. uncinulatum*	Py-2	AB468782	AB468720	AB468720

## Data Availability

Material available to interested researchers upon request.

## References

[1] Carrari E., Aglietti C., Bellandi A., Dibari C., Ferrini F., Fineschi S., Galeotti P., Giuntoli A., Manganelli Del Fa R., Moriondo M. (2022). The management of plants and their impact on monuments in historic gardens: Current threats and solutions. Urban For. Urban Green..

[2] Ciaffi M., Alicandri E., Vettraino A.M., Paolacci A.R., Tamantini M., Tomao A., Agrimi M., Kuzminsky E. (2018). Conservation of Veteran Trees within Historical Gardens (COVE): A case study applied to *Platanus orientalis* L. in Central Italy. Urban For. Urban Green..

[3] Cunha A.R., Soares A.L., Catarino S., Duarte M.C., Romeiras M.M. (2025). Assessing the vulnerability of urban tree species to climate change: The case study of Lisbon gardens. Urban For. Urban Green..

[4] Vettraino A.M., Soulioti N., Matosevic D., Tuğba Doğmuş Lehtijarvi H., Woodward S., Santini A., Luchi N. (2025). Management of fungal diseases of *Platanus* under changing climate conditions: Case studies in urban areas. Urban For. Urban Green..

[5] Antonelli C., Soulioti N., Linaldeddu B.T., Tsopelas P., Biscontri M., Tsoukas C., Paplomatas E., Kuzminsky E., Vettraino A.M. (2024). *Phytophthora nicotianae* and *Ph. mediterranea:* A Biosecurity threat to *Platanus orientalis* and *P.* x *acerifolia* in urban green areas in Greece. Urban For. Urban Green..

[6] Khdiar M.Y., Barber P.A., Hardy G.E.S., Shaw C., Steel E.J., McMains C., Burgess T.I. (2020). Association of *Phytophthora* with declining vegetation in an urban forest environment. Microorganisms.

[7] Tkaczyk M., Sikora K. (2025). First report of the occurrence of *Phytophthora honggalleglyana* in an urban green space in Poland. Urban For. Urban Green..

[8] Vettraino A.M., Matošević D. (2025). Sustainable management strategies for enhancing urban tree health and resilience. Urban For. Urban Green..

[9] Dale A.G., Frank S.D. (2017). Warming and drought combine to increase pest insect fitness on urban trees. PLoS ONE.

[10] Franić I., Cleary M., Aday Kaya A.G., Bragança H., Brodal G., Cech T.L., Chandelier A., Doğmuş-Lehtijärvi T., Eschen R., Lehtijärvi A. (2023). The biosecurity risks of international forest tree seed movements. Curr. For. Rep..

[11] Marcot B.G., Scott P., Burgess T.I. (2023). Multivariate Bayesian analysis to predict invasiveness of *Phytophthora* pathogens. Eco-sphere.

[12] Schiffer-Forsyth K., Frederickson Matika D.F., Hedley P.E., Cock P.J.A., Green S. (2023). *Phytophthora* in horticultural nursery green waste—A Risk to Plant Health. Horticulturae.

[13] Weed A.S., Ayres M.P., Hicke J.A. (2013). Consequences of climate change for biotic disturbances in North American forests. Ecol. Monogr..

[14] Avan M., Palacioğlu G., Sarigül Ertek T., Katircioğlu Y.Z., Bayraktar H., Kaya R., Maden S. (2020). Sugar beet root rot caused by oomycetous pathogens in Turkey and their control by seed treatment. Turk. J. Agric. For..

[15] Ertek T.S., Bayraktar H. (2025). pathogenic oomycota species in walnut nurseries of Bursa and Isparta(Eğirdir) provinces in Türkiye, with a first report of *Phytopythium vexans*, *Phytopythium litorale*, *Pythium aphanidermatum* and *Globisporangium ultimum* on walnut. Physiol. Mol. Plant Pathol..

[16] Kimura M. (1980). A Simple method for estimating evolutionary rates of base substitutions through comparative studies of nucleotide sequences. J. Mol. Evol..

[17] Tamura K., Stecher G., Kumar S. (2021). MEGA11: Molecular evolutionary genetics analysis version 11. Mol. Biol. Evol..

[18] Uzuhashi S., Kakishima M., Tojo M. (2010). Phylogeny of the genus *Pythium* and description of new genera. Mycoscience.

[19] Villa N.O., Kageyama K., Asano T., Suga H. (2006). Phylogenetic relationships of *Pythium* and *Phytophthora* species based on ITS rDNA, Cytochrome Oxidase II and -Tubulin Gene Sequences. Mycologia.

[20] Paul B. (2003). *Pythium glomeratum*, a New species isolated from agricultural soil taken in North-Eastern France, its ITS region and its comparison with related species. FEMS Microbiol. Lett..

[21] Feng H., Chen J., Yu Z., Li K., Li Z., Li Y., Sun Z., Wang Y., Ye W., Zheng X. (2020). Pathogenicity and fungicide sensitivity of *Pythium* and *Phytopythium* Spp. Associated with Soybean in the Huang-Huai Region of China. Plant Pathol..

[22] Rojas J.A., Jacobs J.L., Napieralski S., Karaj B., Bradley C.A., Chase T., Esker P.D., Giesler L.J., Jardine D.J., Malvick D.K. (2017). Oomycete Species Associated with Soybean Seedlings in North America—Part I: Identification and pathogenicity characterization. Phytopathology®.

[23] Derviş S., Özer G., Türkölmez Ş., Çiftçi O. (2020). First report of *Globisporangium heterothallicum* causing root and crown rot of pepper in Turkey. New Dis. Rep..

[24] Moorman G.W., Kang S., Geiser D.M., Kim S.H. (2002). Identification and characterization of *Pythium* species associated with greenhouse floral crops in Pennsylvania. Plant Dis..

[25] Lazreg F., Belabid L., Sánchez J., Gallego E. (2016). Root rot and damping-off of Aleppo pine seedlings caused by *Pythium* spp. in Algerian forest nurseries. J. For. Sci..

[26] Antonelli C., Biscontri M., Tabet D., Vettraino A.M. (2022). The never-ending presence of *Phytophthora* species in Italian nurseries. Pathogens.

[27] Cleary M., Oskay F., Doğmuş H.T., Lehtijärvi A., Woodward S., Vettraino A.M. (2019). Cryptic risks to forest biosecurity as-sociated with the global movement of commercial seed. Forests.

[28] Singh B.K., Delgado-Baquerizo M., Egidi E., Guirado E., Leach J.E., Liu H., Trivedi P. (2023). Climate change impacts on plant pathogens, food security and paths forward. Nat. Rev. Microbiol..

[29] Bregant C., Carloni F., Borsetto G., Delle Donne A.G., Linaldeddu B.T., Murolo S. (2025). Multiple Botryosphaeriaceae and *Phytophthora* species involved in the etiology of Holm Oak (*Quercus ilex* L.) Decline in Southern Italy. Forests.

[30] De Sampaio E Paiva Camilo-Alves C., Da Clara M.I.E., De Almeida Ribeiro N.M.C. (2013). Decline of Mediterranean oak trees and its association with *Phytophthora cinnamomi:* A Review. Eur. J. For. Res..

[31] Jung T., Orlikowski L., Henricot B., Abad-Campos P., Aday A.G., Aguín Casal O., Bakonyi J., Cacciola S.O., Cech T., Chavarriaga D. (2016). Widespread *Phytophthora* infestations in european nurseries put forest, semi-natural and horticultural ecosystems at high risk of *Phytophthora* diseases. For. Pathol..

[32] Vettraino A.M., Barzanti G.P., Bianco M.C., Ragazzi A., Capretti P., Paoletti E., Luisi N., Anselmi N., Vannini A. (2002). Occurrence of *Phytophthora* species in oak stands in Italy and their association with declining oak trees. For. Pathol..

